# Detection and Identification of *Mycoplasmopsis agassizii* in Captive Tortoises with Different Clinical Signs in Italy

**DOI:** 10.3390/ani13040588

**Published:** 2023-02-07

**Authors:** Livio Galosi, Nicola Ridolfi, Cristina Fellini, Igor Pelizzone, Stefano Cusaro, Gianluca Marchetti, Matteo Canonico, Elena Ghelfi, Nicola Di Girolamo, Silvia Preziuso

**Affiliations:** 1 School of Biosciences and Veterinary Medicine, University of Camerino, 62024 Matelica, Italy; 2 Clinica Veterinaria Gaudenzi, 61121 Pesaro, Italy; 3 Clinica Veterinaria Foce, 16129 Genova, Italy; 4 Ambulatorio Veterinario Belvedere, 42123 Reggio Emilia, Italy; 5 Ambulatorio Veterinario Associato XXIII Marzo, 28100 Novara, Italy; 6 Clinica Veterinaria Guidonia, 00012 Guidonia, Italy; 7 Nuovo Ambulatorio Veterinario Artemis, 60019 Senigallia, Italy; 8 Ambulatorio Veterinario Associato Ghelfi Nieddu, 27100 Pavia, Italy; 9 Department of Clinical Sciences, College of Veterinary Medicine, Cornell University, Ithaca, NY 14853, USA

**Keywords:** *Mycoplasmopsis agassizii*, mycoplasmosis, chelonians, PCR, 16S rRNA sequencing

## Abstract

**Simple Summary:**

Some species of turtle and tortoise are vulnerable or declared threatened and pathogens such as *Mycoplasmopsis agassizii* can cause severe declines in their populations. Diagnosis and control of transmissible disease is the key for limiting the spread of pathogens in collection of animals. Sequencing and sharing sequences of mycoplasmas obtained from reptiles is important to improve diagnostic tests and to monitor the species affinity and the cross-species transmission potential of mycoplasmas. This study stresses the important role of practitioners in proper collection and sharing of data for appropriate analysis. The results obtained show that *M. agasizii* is detectable in different specimens and how is important to collect the proper sample to avoid false negative results. Not only animals with upper respiratory diseases should be considered as potentially infected, because *M. agassizii* sequences were found also in asymptomatic animals and in tortoises with stomatitis without respiratory signs. Coinfection with or infection due to other pathogens should be also considered.

**Abstract:**

*Mycoplasmopsis agassizii* causes the Upper Respiratory Tract Disease (URTD) in tortoises. The severity of the disease usually ranges from mild to severe respiratory signs. Animals can recover, die, or become asymptomatic carriers and are source of infection for other tortoises. This study describes (i) the clinical history and the results obtained in ten years of diagnostic PCR activity for detecting *M. agassizii* in different species of captive tortoises in Italy, and (ii) the phylogenetic analysis of the 16S rRNA gene sequences of *M. agassizii*. A total of 26.0% out of 169 samples resulted positive by PCR and 32 out of 75 (42.7%) animals with symptoms were positive. Sequences ob-tained from the PCR products were conserved, differed from the sequence of the *M. agassizii* type strain PS6, and were identical to many *M. agassizii* sequences deposited in databases. In particular, the sequences were identical or very similar to sequences obtained previously from tortoises in It-aly. Since samples collected from different anatomical sites resulted positive, it is suggested that pools of conjunctival, nasal and oral swabs are tested for diagnostic purpose in both symptomatic and asymptomatic animals.

## 1. Introduction

*Mycoplasma agassizii*, recently renamed *Mycoplasmopsis agassizii* [1,2], was discovered in a causal relationship with upper respiratory tract disease (URTD) in desert [3] and gopher tortoises [4]. Mycoplasmosis has since become one of the most extensively characterized infectious diseases of chelonians, and has been detected in many regions of the USA and Europe [5,6,7,8,9]. Clinical signs of Mycoplasmosis in tortoises include palpebral edema, conjunctivitis, and nasal and ocular discharges, although subclinical infection may occur [8]. Although *M. agassizii* typically affects the nasal cavity and the upper respiratory tract, pneumonia is occasionally seen. Mortality and morbidity due to *M. agassizii* infection in tortoises is poorly understood. In some outbreaks, mortality can reach 50%, while in other instances the bacteria are present in the population in a subclinic and chronic form, with tortoises acting as carriers for long time. Different diagnostic tests have been developed for detecting *M. agassizii* or *M. agassizii* antibodies in wild or captive chelonians [8]. Although ELISA tests can be useful to evaluate the exposure of animals to *M. agassizii* by quantifying specific antibodies in plasma and serum, PCR has many advantages for the diagnosis of mycoplasmosis in tortoises, including high specificity and rapid detection. Although PCR does not provide information about the viability of the mycoplasmas at the time of testing, sequencing PCR products provides useful information about genetic variability and new species spread. The most common samples collected and tested for detecting *M. agassizii* using PCR are nasal lavage samples and oral swabs [5,6,7,8,9,10], although comparative studies to identify the best specimen for *M. agassizii* detection via PCR are lacking.

The aims of this study were (i) to investigate *M. agassizii* in samples collected from chelonians during veterinary consultations in Italy and submitted to the laboratory diagnostic service over ten years, and (ii) to study the genetic variability of the 16S rRNA gene of *M. agassizii* and of other mycoplasmas of reptiles similar to, but different than, *M. agassizii*.

## 2. Materials and Methods

A total of 169 samples collected from 169 tortoises and one turtle were included in this study. All chelonians were captured and managed as pet animals by the owners. The samples were collected between 2009 and 2018 from chelonians with and without clinical signs of URTD by 30 different veterinarians located in seven administrative regions in Northern and Central Italy during veterinary consultations. The samples were shipped to the laboratory of molecular diagnosis of the University of Camerino, where DNA extraction and PCR were carried out. Clinical data were collected using a form that veterinarians voluntary filled in. DNA was obtained from the swab samples or from 25 mg of tissue samples using the Genomic DNA Isolation Kit (Norgen Biotek Corp., Thorold, ON, Canada) according to the manufacturer’s instructions, with a few modifications. When more than one swab was collected from the same animal, the swabs were pooled for DNA extraction. DNA was eluted from the silica membranes by adding 100 µL of elution buffer warmed at 55 °C and by centrifuging at 6000× *g* for 2 min and at 13,000× *g* for 5 min. The silica membranes were moved to another 1.5 mL collection tube and the elution step was repeated as noted above to obtain a second aliquot of DNA. The first aliquot of DNA was immediately used for PCR analysis or stored at 4 °C for a maximum of 48 h, and then conserved at −20 °C. The second aliquot of DNA was immediately stored at −20 °C. Control of DNA contamination was carried out using a sample of PCR grade water every 5 clinical samples. PCR testing was carried out using a set of primers previously reported by [3] to amplify a sequence found in the V3 variable region of the 16S rRNA gene of *M. agassizii*. In particular, the PCR mixture included 25 µL 2 X Taq PCR Master Mix (Qiagen), 0.5 pM primer Magas-F (5′-CCT ATA TTA TGA CGG TAC TG-3′), 0.5 pM primer Magas-R (5′-TGC ACC ATC TGT CAC TCT GTT AAC CTC-3′), 2 µL DNA, and PCR grade water up to 50 µL final volume. Samples were subjected to denaturation for 3 min at 94 °C, then to 45 cycles of template denaturation for 1 min at 94 °C, primer annealing for 1 min at 55 °C, and extension for 1 min at 72 °C, followed by 7 min at 72 °C. The 576-bp PCR products were visualized in 1.5% agarose gel. Considering that most *M. agassizii* sequences available in GenBank cover the tract included between primers Magas-F and Magas-R, the products obtained from the samples were submitted to Sanger sequencing by an external laboratory for phylogenetic analysis. Both sense and antisense strands were manually checked and edited using the BioEdit 7.2 program. A preliminary analysis of the nucleotide sequences was carried out using BLASTn (https://blast.ncbi.nlm.nih.gov/ (accessed on 9 November 2022)) to detect regions of similarity with sequences included in databases. Sequences were aligned using MUSCLE [11] with all *M. agassizii*, *Mycoplasma testudineum* and *Mycoplasma testudinis* 16S rRNA sequences available in GenBank, and with other mycoplasmas with the higher identity with the sequences obtained (first 100 sequences obtained using BLASTn were selected). The phylogenetic trees were inferred using the MEGA 11 program [12]. The best-fitting nucleotide substitution models were estimated, and the Kimura 2-parameter model [13] with a gamma distribution with invariant sites and bootstrap values based on 1000 repetitions was used. Phylogeny was estimated using the maximum likelihood method.

## 3. Results

Most samples included in this study originated from *Testudo hermanni*, *Testudo graeca*, *Testudo marginata*, *Stigmochelys pardalis*, and *Testudo horsfieldii*, while genus and species of tortoise were not disclosed in 25 samples. Positive results depending on the species are reported in Table 1.

PCR analysis detected DNA of *M. agassizii* in 44 out of 169 (26.0%) samples in 11 species of tortoise. Ten of these samples were from animals with undisclosed clinical history. Twenty-four out of 35 positive animals with disclosed clinical history showed respiratory signs (68.6%), which were associated with stomatitis (*n* = 3), death (*n* = 1), sto-matitis and death (*n* = 1) or stomatitis and conjunctivitis (*n* = 3). Two positive animals were asymptomatic, 8 tortoises had no respiratory signs but showed stomatitis (*n* = 4), sudden death (*n* = 3), conjunctivitis (*n* = 1) (Table 2).

Negative samples were mainly from tortoises with undisclosed clinical history (*n* = 53) or from asymptomatic animals (*n* = 30). In 7 negative animals the diagnostic test for *M. agassizii* was requested on samples from tortoises with a history of soft-carapace, oral neoformation, icterus, anorexia, ataxia, lethargy, or neurologic sign (*n* = 2) (Table 3). 

The specimens submitted to the laboratory for diagnosis were collected from different anatomical sites (Supplementary Table S1). Of 44 samples resulted positive by PCR, 23 were oral swabs, six of which were pooled with nasal swabs and other 6 of which were pooled with nasal and ocular swabs. Three samples were only nasal swabs, 3 were nasal and pharyngeal swabs, 4 were only pharyngeal swabs, and 7 were swabs of un-known origin. Esophageal and oral necrotic tissues resulted positive in a dead animal, such as lung samples collected from 3 dead tortoises. Most negative samples were oral swabs, while a few nasal swabs were negative. 

**Figure 1:**
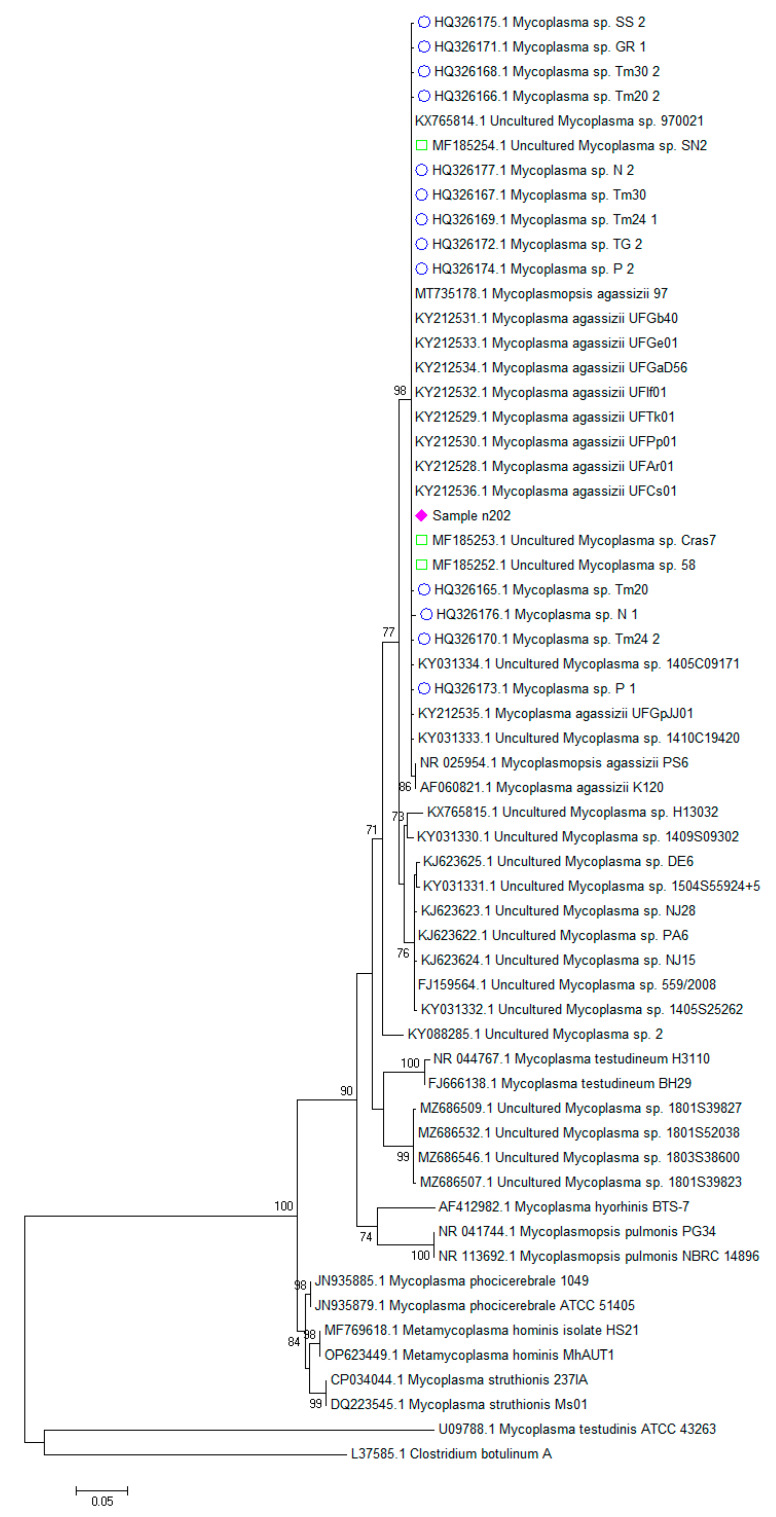
Phylogenetic analysis of the 16S rRNA partial sequence of the samples and of other mycoplasmas. *Clostridium botulinum* strain ATCC 25,763 was also included in the dataset as an outgroup. The evolutionary history was inferred using the maximum likelihood method based on the Kimura-2-parameter model with a gamma distribution with invariant sites and with bootstrap values based on 1000 repetitions. The tree is unrooted. Bootstrap support values above 70% are represented. As the sequences obtained in this study were identical, only a representative sequence was included in the tree and labeled with a diamond (♦). Sequences obtained previously in tortoises in Italy are labeled with a circle (○) [7] and a square (□) [9].

All samples showed the same 16S rRNA sequence, which was identical to most *M. agassizii* sequences deposited in the sequence databases. The sequence was deposited in GenBank with ID number OQ312100. Phylogenetic analysis showed that our samples clustered within a group of mycoplasmas previously detected in tortoises, but they slightly differed from a small group including the *M. agassizii* type strain PS6 (ID NR025954) and from a larger group including uncultured *Mycoplasma* sp. detected in different species of tortoise and turtle (Figure 1). The hosts and countries of origin of the sequences obtained from reptiles and included in the phylogenetic tree are summarized in Supplementary Table S1. Our samples were clearly differed from the group of mycoplasmas detected in pythons of different genera [14] and from *M. testudinis*. All these mycoplasmas detected in reptiles clustered in a main branch separated from those detected in non-reptile hosts. 

The hosts and countries of origin of the sequences obtained from reptiles and in-cluded in the phylogenetic tree are summarized in the Supplementary Table S2. Our samples were clearly different than the group of mycoplasmas detected in pythons of different genera [14] and from *M. testudinis*. All these mycoplasmas detected in reptiles clustered in a main branch separated from those detected in non-reptile hosts. 

Considering the sequence of the type strain PS6 (ID NR025954) as the reference sequence, the primary nucleotidic changes observed among *M. agassizii* strains and other related *Mycoplasma* sp. sequences were A559T and G715A (Table 4). 

Other few scattered single changes were found in some sequences clustered within the same branch. Other relevant changes were found in a small group of *Mycoplasma* sp. sequences clustered in a separate branch at positions 545, 559, 599, 601, 621, 689, 715, 790, 794, 798, 946 and 947. Based on the sequences available in the sequence databases and on the BLASTn results, the organisms most related to these unclassified sequences are *M. agassizii*, although significant differences in the 16S rRNA sequences justified the cluster-ing in a separate group.

## 4. Discussion

This study describes the clinical and laboratory findings in tortoises infected by *M. agassizii* in Italy between 2009 and 2018. *M. agassizii* was detected by PCR in 26.0% of 169 samples submitted to the laboratory for diagnosis. However, if samples from asymptomatic animals or from animals with undisclosed clinical history are not considered, a total of 42.7% positive samples were obtained from 75 tortoises showing different clinical signs. This percentage is higher and lower than previously found in free-ranging and rescued tortoises in Italy, respectively by [7,9]. However, the results of these studies should be compared with caution because of some inherent differences in their study design. Indeed, in the first study wild tortoises captured and recovered in a wildlife center mainly because of traumas or other injuries were considered [7]. In the second study, free-ranging and rescued *Testudo h. hermanni* were collected [9]. In both studies, oral and cloacal swabs were collected for multiple analysis, while nasal samples were not collected. On the contrary, in the present study only samples collected from pet tortoises during veterinary visits were included, and many nasal swabs were tested.

The results obtained in this study confirm that *M. agassizii* is not species specific, indeed it has been found in 11 species of tortoise. One of the limits of this study is that it is based on retrospective sampling and collection of clinical history. Unfortunately, several data were missing or undisclosed, stressing the important role of practitioners in proper collection and sharing of data for appropriate analysis. Interestingly, *M. agassizii* has been found not only in animals with upper respiratory diseases, as expected, but also in animals with stomatitis, with a history of sudden death or asymptomatic. On the contrary, *M. agassizii* has been not found in tortoises with upper respiratory diseases, suggesting that other pathogens were the causative agents. Even if viral diseases are suspected, it is suggested to test the animals also for *M. agassizii*, which can be found sometimes as co-infecting pathogen in atypical clinical presentations, in order to choose the proper therapy and management of collections of tortoises.

In absence of comparative studies, it is not known the exact route and dynamic of excretion of *M. agassizii* in infected tortoise. It is possible that some negative results obtained in symptomatic animals were false negative results due to fails in sampling. Mycoplasma colonize the ventrolateral mucosal surface of the upper respiratory tract in tortoises [4], thus oral swabs can result in false negatives. In our experience, the same *M. agassizii* infected tortoise resulted sometimes positive by testing the nasal swab and negative by testing the oral swab and *vice versa*. Similar observations have been made by other authors, which tested 23 chelonians and found *M. agassizii* in both oral swab and nasal wash in 11 chelonians, while 8 chelonians were only positive in the swab sample, and 4 chelonians were only positive in nasal washes [15].Thanking to the high sensibility and specificity of the PCR protocol, it is possible to test pool of samples from the same animal to decrease the probability of false positive results due to bias in sampling. When possible, samples from ocular, nasal and oro-pharyngeal swabs should be submitted for *M. agassizii* diagnosis. Analysis of these samples in pool allow to reduce the costs of analysis and optimize the diagnosis. Lung samples and oral lesions are the best specimens from dead animals in addition to nasal and oral swabs. Analysis of results and exchange of information between practitioners and clinical microbiologists is important to continuously improve the diagnostic protocols and sampling procedures.

In silico analysis of the sequences obtained in this study confirmed that all PCR products were from *M. agassizii*. Sequences of bacterial 16S rRNA are usually conserved and are at the basis of taxonomic classification, although some changes can be observed in different strains. The samples tested in this study showed a highly conserved 16S rRNA sequence, confirming that *M. agassizii* are low variable bacteria [16]. However, the alignment with sequences obtained from the databases showed that slight changes were present at specific nucleotidic positions, suggesting the existence of variants. All sequences found in this study, and most sequences deposited in the databases, were almost identical and differed from the sequence of *M. agassizii* type strain PS6. Other groups of sequences deposited as *Mycoplasma* sp. showed higher identity to *M. agassizii* sequences, although several nucleotidic changes were present at specific positions. Further sequencing of the 16S rRNA of additional strains could improve the knowledge of the epidemiology and pathogenicity of these different strains of mycoplasmas.

## 5. Conclusions

*Mycoplasmopsis agassizii* is widely spread in several species of captive tortoises in Italy affected by different diseases. The strains show a unique nucleotidic sequence of 16S rRNA and belong to a large group of *M. agassizii*, which slight differs from the *M. agassizii* type strain PS6. Sequencing and sharing sequences of mycoplasmas obtained from reptiles is important to improve diagnostic tests and to monitor the species affinity and the cross-species transmission potential of mycoplasmas. In the absence of an ultimate definition of the best specimens to collect for the diagnosis of *M. agassizii* in turtle and tortoise, it is suggested that a pool of conjunctival, nasal, and oro-pharyngeal swabs, and lung samples from dead animals, be analyzed via PCR. 

## Figures and Tables

**Table 1:** Species of tortoise tested for *Mycoplasmopsis agassizii* by PCR.

Tortoises	Positive	Negative	Total
*Aldabrachelys gigantea*	1	0	1
*Astrochelys radiata*	1	2	3
*Centrochelys sulcata*	0	7	7
*Chelonoidis carbonarius*	0	1	1
*Geochelone* sp.	1	0	1
*Geochelone elegans*	2	0	2
*Kinixys belliana*	0	1	1
*Stigmochelys pardalis*	9	7	16
*Testudo* sp.	1	0	1
*Testudo graeca*	5	14	19
*Testudo graeca nabeulensis*	1	0	1
*Testudo hermanni*	6	52	58
*Testudo hermanni boettgeri*	0	3	3
*Testudo horsfieldii*	6	9	15
*Testudo kleinmanni*	2	2	4
*Testudo marginata*	3	8	11
n.a.	6	19	25
Total	44	125	169

**Table 2:** Clinical signs observed by veterinarians in different species of tortoise resulted positive for *Mycoplasmopsis agassizii* by PCR at the time of sampling. Legend: R = respiratory, C = conjunctivitis, S = stomatitis, D = death, No = asymptomatic, n.a. = not available.

	Clinical Signs										
Tortoises	Respiratory (R)	Cconjunctivitis (C)	Stomatitis (S)	Death (D)	R+S	R+D	R+C+S	R+S+D	No	n.a.	TOTAL
*Aldabrachelys gigantea*	1										1
*Astrochelys radiata*			1								1
*Centrochelys sulcata*											0
*Chelonoidis carbonarius*											0
*Geochelone* sp.						1					1
*Geochelone elegans*					1					1	2
*Kinixys belliana*											0
*Stigmochelys pardalis*	3		1		1		2		1	1	9
*Testudo* sp.										1	1
*Testudo graeca*	5										5
*Testudo graeca nabeulensis*										1	1
*Testudo hermanni*	3		1	1						1	6
*Testudo hermanni boettgeri*											0
*Testudo horsfieldii*			1		1		1			3	6
*Testudo kleinmanni*				1					1		2
*Testudo marginata*	3										3
n.a.	1	1		1				1		2	6
TOTAL	16	1	4	3	3	1	3	1	2	10	44

**Table 3:** Clinical signs observed in different species of tortoise resulted negative for *Mycoplasmopsis agassizii* by PCR. Legend: R = respiratory, C = conjunctivitis, S = stomatitis, D = death, No = asymptomatic, n.a. = not available, Other = showing diseases different than those reported in the previous columns.

	Clinical Signs													
Tortoises	Respiratory (R)	Cconjunctivitis (C)	Stomatitis (S)	R+S	R+ Death (D)	R+C	C+S	C+D	S+D	R+C+S	No	n.a.	Other	TOTAL
*Aldabrachelys gigantea*														0
*Astrochelys radiata*												2		2
*Centrochelys sulcata*	2										2	2	1	7
*Geochelone* sp.														0
*Chelonoidis carbonarius*												1		1
*Geochelone elegans*														0
*Kinixys belliana*												1		1
*Stigmochelys pardalis*	1					1					1	4		7
*Testudo* sp.														0
*Testudo hermanni boettgeri*												3		3
*Testudo graeca*	3		1								1	7	2	14
*Testudo graeca nabeulensis*														0
*Testudo hermanni*	4	3	1	2	1		2	1	1		18	17	2	52
*Testudo horsfieldii*	1	1								1		5	1	9
*Testudo kleinmanni*												2		2
*Testudo marginata*						1					6	1		8
n.a.	4		1	3							2	8	1	19
TOTAL	15	4	3	5	1	2	2	1	1	1	30	53	7	125

**Table 4:** Nucleotide variations in the 16S rRNA sequences representative of strains and samples. Sample #202 is representative of all sequences obtained in this study. Sequences are aligned with reference to the type strain PS6 (ID NR025954). Dots (.) indicate sequence identity.

	Nucleotide Position												
	545	559	599	601	621	689	715	790	794	798	946	947	970
NR025954 *Mycoplasmopsis agassizii* PS6	G	A	C	A	A	T	G	G	C	G	C	T	G
MT735178.1 *Mycoplasmopsis agassizii* 97	.	T	.	.	.	.	A	.	.	.	.	.	.
Sample #202	.	T	.	.	.	.	A	.	.	.	.	.	.
KY031330.1 Uncultured *Mycoplasma* sp. 1409S09302	A	T	T	.	.	.	A	A	T	.	T	C	.
KY031331.1 Uncultured *Mycoplasma* sp. 1504S55924 + 5	A	T	T	G	G	C	A	A	T	A	T	C	.
KJ623624.1 Uncultured *Mycoplasma* sp. NJ15	A	C	T	G	G	C	A	A	T	.	T	C	C
KJ623625.1 Uncultured *Mycoplasma* sp. DE6	A	C	T	G	G	C	A	A	T	A	T	C	C

## Data Availability

Sequences obtained from this study were deposited in open databases (GenBank); codes of sequences used for the phylogenetic analysis are reported in Figure 1 and the sequences are freely available in open databases (e.g., GenBank).

## Supplementary Materials

The following supporting information can be downloaded at: https://www.mdpi.com/article/10.3390/ani13040588/s1, Table S1: Types of specimens collected from tortoises and tested for *M. agassizii*; Table S2: Hosts and countries from which the strains used in the dataset for the phylogenetic analysis were obtained.
