# Supplementary material for: Detection and Identification of Mycoplasmopsis agassizii in Captive Tortoises with Different Clinical Signs in Italy
Source: Animals (Basel). 2023 Feb 7;13(4):588. doi: 10.3390/ani13040588 (PMC9951701; doi:10.3390/ani13040588)
Supplement: Supplementary file 1 [file animals-13-00588-s001.zip › animals-2167672-supplementary.pdf]

Table S1. Types of specimens collected from tortoises and tested for *M. agassizii* by PCR. Legend: n.a. = information was not available.

Positive samples		Negative samples	
**Sample number**	**Specimen**	**Sample number**	**Specimen**
1	pharyngeal swab	2	oral swab
19	nasal and pharyngeal swabs	3	oral swab
20	oral swab	4	oral swab
22	nasal, oral and ocular swabs	5	oral swab
28	nasal, oral and ocular swabs	6	oral swab
29	swab	7	oral swab
32	oral swab	8	oral swab
35	oral swab	9	oral swab
53	oral swab	10	oral swab
62	nasal, oral and ocular swabs	11	oral swab
70	oral swab	12	oral swab
77	swab	13	oral swab
83	pharyngeal swab	14	oral swab
86	lung, trachea and tongue	15	oral swab
87	nasal, oral and ocular swabs	16	oral swab
88	oral swab	17	laryngeal tracheal swab
91	lung and SNC	18	oral swab
98	nasal swab	21	oral swab
99	nasal swab	25	n.a.
101	oral swab	26	n.a.
112	nasal, oral and ocular swabs	30	n.a.
146	swab	31	n.a.
155	oro-pharyngeal swab	33	pharyngeal swab
156	swab	34	pharyngeal swab
157	nasal and oral swabs	36	oral swab
160	swab	38	oral swab
161	lung	39	oral swab
162	oral swab	40	oral swab
164	nasal and pharyngeal swabs	41	oral swab
165	pharyngeal swab	42	oral swab
168	oral swab	43	oral swab
170	nasal and pharyngeal swabs	44	oral swab
171	nasal and oral swabs	45	oral swab
172	nasal and oral swabs	46	oral swab
174	nasal and oral swabs	47	oral swab
175	nasal and oral swabs	48	oral swab
176	oral swab	49	oral swab
178	swab	50	oral swab
179	nasal, oral and ocular swabs	51	oral swab
181	nasal swab	52	oral swab
185	nasal and oral swabs	54	oral swab
186	pharyngeal swab	55	oral swab
202	Esophageal and oral necrotic tissue	56	oral swab

205	swab	57	oral swab
		58	n.a.
		59	oral swab
		60	oral and ocular swabs
		61	oral and ocular swabs
		63	n.a.
		64	n.a.
		65	oral swab
		66	n.a.
		67	n.a.
		68	pharyngeal swab
		69	oral swab
		71	nasal and ocular swabs
		72	n.a.
		73	n.a.
		74	n.a.
		75	n.a.
		76	n.a.
		78	ocular swab
		79	pharyngeal swab
		80	pharyngeal swab
		81	oral swab
		82	n.a.
		84	oral swab
		85	n.a.
		89	n.a.
		90	n.a.
		100	pharyngeal swab
		104	oral swab
		109	oral swab
		110	oral swab
		111	oral swab
		113	pharyngeal swab
		114	pharyngeal swab
		115	pharyngeal swab
		116	n.a.
		117	pharyngeal swab
		118	pharyngeal swab
		119	pharyngeal swab
		120	n.a.
		121	n.a.
		127	oral swab
		128	oral neoformation
		129	nasal and pharyngeal swabs
		130	n.a.
		131	n.a.
		132	n.a.
		138	n.a.

	139	oral swab
	140	n.a.
	141	n.a.
	143	nasal and oral swabs
	148	n.a.
	149	oro-pharyngeal swab
	150	laryngeal esophageal swab
	151	oral and ocular swab
	152	n.a.
	154	oro-pharyngeal swab
	158	nasal swab
	159	oral swab
	163	n.a.
	166	oral swab
	167	pharyngeal swab
	173	nasal and oral swabs
	177	n.a.
	182	nasal and oral swabs
	183	nasal and oral swabs
	184	n.a.
	188	oral swab
	189	oral swab
	190	oral swab and tissue
	191	nasal and oral swabs
	192	oral and ocular swab
	193	n.a.
	194	n.a.
	195	n.a.
	196	oral and ocular swab
	197	oral, ocular and cloacal swabs
	203	nasal, ocular, oral and cloacal swabs
	204	oral, ocular and cloacal swab
	206	oral swab
	207	n.a.
	208	n.a.

Table S2. Hosts and countries from which the strains used in the dataset for the phylogenetic analysis were obtained. Only strains obtained from reptils are included in this table.

Strain	Host	Country
AF060821.1_Mycoplasma agassizii_K120	Tortoises	USA
FJ159564.1_Uncultured Mycoplasma sp._559/2008	*Terrapene ornata ornata*	Hungary
FJ666138.1_Mycoplasma testudineum_BH29	*Gopherus agassizii*	USA
HQ326165.1_Mycoplasma sp. Tm20	*Testudo marginata*	Italy
HQ326166.1_Mycoplasma sp. Tm20_2	*Testudo marginata*	Italy
HQ326167.1_Mycoplasma sp. Tm30	*Testudo marginata*	Italy
HQ326168.1_Mycoplasma sp. Tm30_2	*Testudo marginata*	Italy
HQ326169.1_Mycoplasma sp. Tm24_1	*Testudo marginata*	Italy
HQ326170.1_Mycoplasma sp. Tm24_2	*Testudo marginata*	Italy
HQ326171.1_Mycoplasma sp. GR_1	*Testudo graeca*	Italy
HQ326172.1_Mycoplasma sp. TG_2	*Testudo graeca*	Italy
HQ326173.1_Mycoplasma sp. P_1	*Testudo marginata*	Italy
HQ326174.1_Mycoplasma sp. P_2	*Testudo marginata*	Italy
HQ326175.1_Mycoplasma sp. SS_2	*Testudo graeca*	Italy
HQ326176.1_Mycoplasma sp. N_1	*Testudo marginata*	Italy
HQ326177.1_Mycoplasma sp. N_2	*Testudo marginata*	Italy
KJ623622.1_Uncultured Mycoplasma sp._PA6	*Glyptemys muhlenbergii*	USA
KJ623623.1_Uncultured Mycoplasma sp._NJ28	*Clemmys guttata*	USA
KJ623624.1_Uncultured Mycoplasma sp._NJ15	*Terrapene carolina carolina*	USA
KJ623625.1_Uncultured Mycoplasma sp._DE6	*Glyptemys muhlenbergii*	USA
KX765814.1_Uncultured Mycoplasma sp._970021	*Indotestudo forstenii*	USA
KX765815.1_Uncultured Mycoplasma sp._H13032	*Leucocephalon yuwonoi*	USA
KY031330.1_Uncultured Mycoplasma sp._1409S09302	*Heosemys grandis*	UK
KY031331.1_Uncultured Mycoplasma sp._1504S55924+5	*Trachemys scripta elegans*	Belgium
KY031332.1_Uncultured Mycoplasma sp._1405S25262	*Trachemys scripta scripta*	Germany
KY031333.1_Uncultured Mycoplasma sp._1410C19420	*Geochelone elegans*	Switzerland
KY031334.1_Uncultured Mycoplasma sp._1405C09171	*Testudo horsfieldii*	Switzerland
KY088285.1_Uncultured Mycoplasma sp._2	*Batagur affinis edwardmolli voucher Ba5*	Cambodia
KY212528.1_Mycoplasma agassizii_UFAr01	*Astrochelys radiata*	USA
KY212529.1_Mycoplasma agassizii_UFTk01	*Testudo kleinmanni*	USA
KY212530.1_Mycoplasma agassizii_UFPp01	*Pyxis planicauda*	USA
KY212531.1_Mycoplasma agassizii_UFGb40	*Gopherus berlanderi*	USA
KY212532.1_Mycoplasma agassizii_UFIf01	*Indotestudo forstenii*	USA
KY212533.1_Mycoplasma agassizii_UFGe01	*Geochelone elegans*	USA
KY212534.1_Mycoplasma agassizii_UFGaD56	*Gopherus agassizii*	USA
KY212535.1_Mycoplasma agassizii_UFGpJJ01	*Gopherus polyphemus*	USA
KY212536.1_Mycoplasma agassizii_UFCs01	*Centrochelys sulcata*	USA
MF185252.1_Uncultured Mycoplasma sp._58	*Testudo hermanni*	Italy

MF185253.1_Uncultured Mycoplasma sp._Cras7	*Testudo hermanni*	Italy
MF185254.1_Uncultured Mycoplasma sp._SN2	*Testudo hermanni*	Italy
MT735178.1_Mycoplasmopsis agassizii_97	*Stigmochelys pardalis*	Austria
MZ686507.1_Uncultured Mycoplasma sp._1801S39823	*Simalia boeleni*	Germany
MZ686509.1_Uncultured Mycoplasma sp._1801S39827	*Simalia boeleni*	Germany
MZ686532.1_Uncultured Mycoplasma sp._1801S52038	*Python brongersmai*	Germany
MZ686546.1_Uncultured Mycoplasma sp._1803S38600	*Python regius*	Germany
NR_025954.1_Mycoplasmopsis agassizii_PS6	*Gopherus agassizii*	USA
NR_044767.1_Mycoplasma testudineum_H3110	*Gopherus agassizii*	USA
U09788.1 Mycoplasma testudinis_ATCC 43263	*Testudo graeca*	USA
